# Nanoparticle-Based Combination Therapy for Melanoma

**DOI:** 10.3389/fonc.2022.928797

**Published:** 2022-06-28

**Authors:** Hongbo Chen, Kai Hou, Jing Yu, Le Wang, Xue Chen

**Affiliations:** ^1^ Department of Plastic and Cosmetic Surgery, Tongji Hospital, Tongji Medical College, Huazhong University of Science and Technology, Wuhan, China; ^2^ Department of Nephrology, Tongji Hospital, Tongji Medical College, Huazhong University of Science and Technology, Wuhan, China

**Keywords:** nanoparticles, combined therapy, melanoma, chemotherapy, immunotherapy, photodynamic therapy

## Abstract

Melanoma is a cutaneous carcinoma, and its incidence is rapidly increasing with every year. The treatment options for melanoma have been comprehensively studied. Conventional treatment methods (e.g., radiotherapy, chemotherapy and photodynamic therapy) with surgical removal inevitably cause serious complications; moreover, resistance is common. Nanoparticles (NPs) combined with conventional methods are new and promising options to treat melanoma, and many combinations have been achieving good success. Due to their physical and biological features, NPs can help target intended melanoma cells more efficiently with less damage. This creates new hope for a better treatment strategy for melanoma with minimum damage and maximum efficacy.

## Introduction

### Overview of Melanoma

Melanoma is a malignant tumour originating from cells called melanocytes. The melanocytes can synthesise melanin, which imparts colour to the skin (1). They are located in the deepest layer of the epidermis and usually protect us from sunburn. Excessive exposure to ultraviolet (UV) radiation increases the occurrence of melanoma (2–5).

Melanoma can form from a congenital or *de novo* nevus. It can appear anywhere on the body, mainly on the parts that are more exposed to the sun, such as the face, leg, arm, and body (6). Low exposed parts have less possibilities to develop the disease, such as the feet.

Although the clinical appearance of a pigmented or unpigmented (amelanotic) lesion with sudden change can be clues for diagnosis, histological staining and classification can predict the prognosis (7). The prognosis is very poor when there is distant metastasis (8, 9).

### Epidemiology of Melanoma

Although melanoma accounts for only 1% of all skin cancers, it causes the highest number of deaths among all skin cancers (10). In the world, 324,635 new cases of melanoma were diagnosed in 2020 and US constituted 106,000 new cases, which is nearly one third. Among all these new cases, 57,043 were dead around the world (11). It was the fifth most common cancer (excluding non-melanoma skin cancer). The incidence is slightly higher in men and usually occurs in people aged 55−84 years (10). It has increased rapidly during the last 50 years in the US, and the incidence varies with age, sex, and geographical location (12–15). There are some factors influencing the incidence of melanoma, and these are important for preventing the disease.

First one is Ethnicity: It is reported that fair-skinned Caucasian populations are more likely to be affected by melanoma (15, 16). This is because the absorption of UVB (Ultraviolet B-rays) radiation is 50% higher in dark-skinned people and results in lower cell death and malignant transformation (17). Geography also plays an important role: “Latitude gradient” is a phenomenon described by Lancaster; it shows a higher incidence of melanoma with increasing proximity to the equator (18–20). The third factor is Age: Elderly people are more susceptible to melanoma, and the highest incidence is among those aged 70−80 years (21). The last factor is sex: Men are more likely to develop melanoma in part because it is androgen driven (22).

The mortality rate due to melanoma is higher in men than in women worldwide (23), with the proportion of annual deaths in the US being 4.0:100,000 in men compared to 1.7:100,000 in women (24). The peak mortality rate is in the age group of 75−84 years and declines thereafter (24).

### Subtypes of Melanoma

There are four subtypes of melanoma based on the tissue from which the primary tumour arises. The major subtype is cutaneous melanoma (CM), which usually accompanies the *BRAF*(v-raf murine sarcoma viral oncogene homolog B1), *RAS* (Rat sarcoma virus), *NF1*(Neurofibromatosis 1), and *TWT*(triple wild-type) mutations (25, 26). Acral melanoma (AM), a distinct form that originates in the palms, soles, and nail beds, is associated more with the *TWT* mutation (27).

Mucosal melanoma (MM), the rarest subtype, has a progressive onset in clinics. Compared to other mutations, loss of *PTEN* mutation or activation of *KIT* and *CCND1* or *CDK4* are more common in MM (28). The last subtype is uveal melanoma (UM), which develops from melanocytes in the uveal tract of the eye with predominantly *GNAQ* and *GNA11* mutations (29). In recent times, the cutaneous melanoma subtype has shown a better prognosis.

Melanoma divided according to the driving mutations also has four subtypes: *BRAF*-mutant, *NRAS*-mutant, *NF1*-loss, and *TWT*. The *BRAF*, *NRAS*, and *NF1* mutations activate the mitogen-activated protein kinase (MAPK) pathway and usually occur during the early stage of tumour (30).

Prognosis biomarkers and related treatments are under investigation, and most data comes from European countries. More sequencing data is needed from non-European countries in order to have a blueprint of the whole mutated genes from different races.

### Molecular Pathology of Melanoma

Melanocytic neoplasms include benign lesions named melanocytic naevi, to malignant types termed melanomas, or even progressive stage, termed metastatic melanoma. Melanocytes, from which all melanocytic neoplasms originate, can grow out of control with excessive UV damage. There are almost 3 billion melanocytes in the skin of an average human being (31). Normally, melanocytes divide less than twice a year (32), and when the adjacent keratinocytes are damaged by UV radiation, melanocytes secrete melanin to protect the keratinocyte nucleus from DNA damage (33).

Melanocytic naevi are benign proliferation of melanocytes and are normally unlikely to progress to melanoma. A common naevus is usually derived from the *BRAF^V600E^* mutation (34). Dysplastic naevi are an intermediate stage between naevi and melanoma (35, 36). The mutations that activate MAPK signalling and *TERT* promoter mutations or hemizygous alterations of *CDKN2A* are drivers of dysplastic naevi (34). Multiple dysplastic naevi are considered high risk factors for melanoma (35, 37)and they are obtaining *NRAS* mutation except *BRAF^V600E^* (34). *BRAF*, *NRAS*, and *TERT* mutations have been reported as the most likely mutations in melanoma *in situ* (38, 39). As MARK signalling plays an important role in melanoma, *TERT* mutations can lead to progression to invasive melanoma. *ARID2* and *ARID1A* are two genes that also influence the progression (34). For melanoma to metastasise to distant organs, the key is activation of genes in the WNT signalling pathway (40–42).

### Treatment Strategies for Melanoma

Each tumour has its own feature, such as location, stage, and genetic profile; thus, the treatment strategy can be quite varied. The treatment options include surgical resection, chemotherapy, radiotherapy, photodynamic therapy (PDT), immunotherapy, or targeted therapy. For stage I–IIIB melanoma, surgical resection is the first choice (43–45) followed by targeted therapy and combined immunotherapy (44, 46). In case of distant metastasis, chemotherapy is necessary (45, 46). Chemotherapy is a good choice for advanced melanoma, and dacarbazine (DTIC) is the key agent (47); however, temozolomide can also be a good choice (47). BRAF inhibitors and MEK inhibitors were a breakthrough in the treatment of melanoma and around 15–20% of tumors finally show primary resistance to this treatment, and moreover, some patients even develop acquired resistance to therapy. The causes of drug resistance can be reactivation of MAPK pathway due to changes in the BRAF protein or interactions of BRAF inhibitors with wild type BRAF proteins (48). Electrochemotherapy is used as a tunnel to deliver drugs to the cells through an electric pulse (49, 50). No adverse effects have been reported, and 85% of patients responded to the treatment (49). However, there are few reports about this treatment, and more research is necessary. Some researchers combined PDT and DTIC, and it successfully reduced the resistance in metastatic melanoma (51).

Since the 19^th^ century, researchers have found that the immune system plays an essential role in tumour metastasis (52). T cells can recognise the tumour cell antigens and destroy them. Interferon α-2b was reported to reduce the recurrence of melanoma and thus increase the patient survival (53); however, few patients responded to the treatment (54). Following this, many more agents to treat melanoma were approved by the United States Food and Drug Administration, including Interleukin-2 in 1998 (55), Ontak (Treg inhibition) in 1999 (56), Peginterferon α-2b (Peg-IFN) and Ipilimumab (Cytotoxic T lymphocyte-associated antigen 4 (CTLA-4) blockade) in 2011 (53, 57),, Nivolumab (Programmed cell death protein 1 (PD-1)/PD-1 ligand (PD-L1) blockade) in 2014 (58), and Talimogene laherparepvec as the first oncolytic virus therapy in 2015 (59). Other immune therapies are under investigation, such as Adoptive T-cell therapy (NCT03060356 and NCT02830724).

Bio-chemotherapy is the combination of chemotherapy and immunotherapy, and it did not show an overall improvement in survival (60). As mentioned earlier, melanoma is triggered by a bunch of gene mutations involved in different signalling pathways. *BRAF* inhibitors, including vemurafenib in 2011 (61), dabrafenib in 2013 (62), and encorafenib, which is currently undergoing clinical trials, are some choices to increase the overall survival; however, these agents have severe adverse effects. *MEK* (Mitogen-activated protein kinase kinase)inhibitors, tametinib in 2013 (63), cobimetinib in 2015 (64) are always administered in combination with *BRAF* inhibitors to treat melanoma. *VEGF* (Vascular endothelial growth factor)inhibitors (65), PI3K-AKT-mTOR pathway inhibitors (66), cyclin-dependent kinase inhibitors (67), and ErbB4 inhibitors (68) also target different signalling pathways to inhibit the growth of melanoma cells.

## Overview of Nanotechnology in Cancer

Nanotechnology is used to deliver medications to the cancer cells *via* nanoparticles (NPs) with minimum adverse effects (69). It is also used in cancer diagnosis, gene therapy, biomarker making, targeted therapy, and imaging. NPs, due to their biological features, can target the cancer cells precisely with little damage to the healthy organs (70). Nanocarriers including liposomes, carbon nanotubes, polymeric micelles, dendrimers, and quantum dots are increasingly widely used in cancer treatment. Liposomes are nanocarriers composed of lipid bilayers encapsulating an aqueous core. The structure of carbon nanotubes is a hollow sphere, ellipsoid and/or tube. Polymeric micelles are nanoscopic core structures derived from amphiphilic block copolymers. Dendrimers are formed by a small atom or group of atoms surrounded by dendrons, which can be classified as nanosized, symmetrical molecules. Quantum dots are nanoscale crystals which are made by human to transport electrons. All of them are applied in different fields and help to delivery drugs.

Some NPs are used to diagnose cancer, like MoS2 nanomaterials used as a platform material in bladder cancer diagnosis (71);NPs as biomarker in the diagnosis of early ovarian cancer (72).And some NPs are used to treat diseases, like NPs based on ROS even can be used in the treatment of myocardial ischemia reperfusion injury (73):NPs based on rheumatoid arthritis microenvironment can be a new direction for rheumatoid arthritis treatment (74). Different NPs based on different physical properties can be used in different ways.

### NPs for Drug Delivery

**Figure 1:**
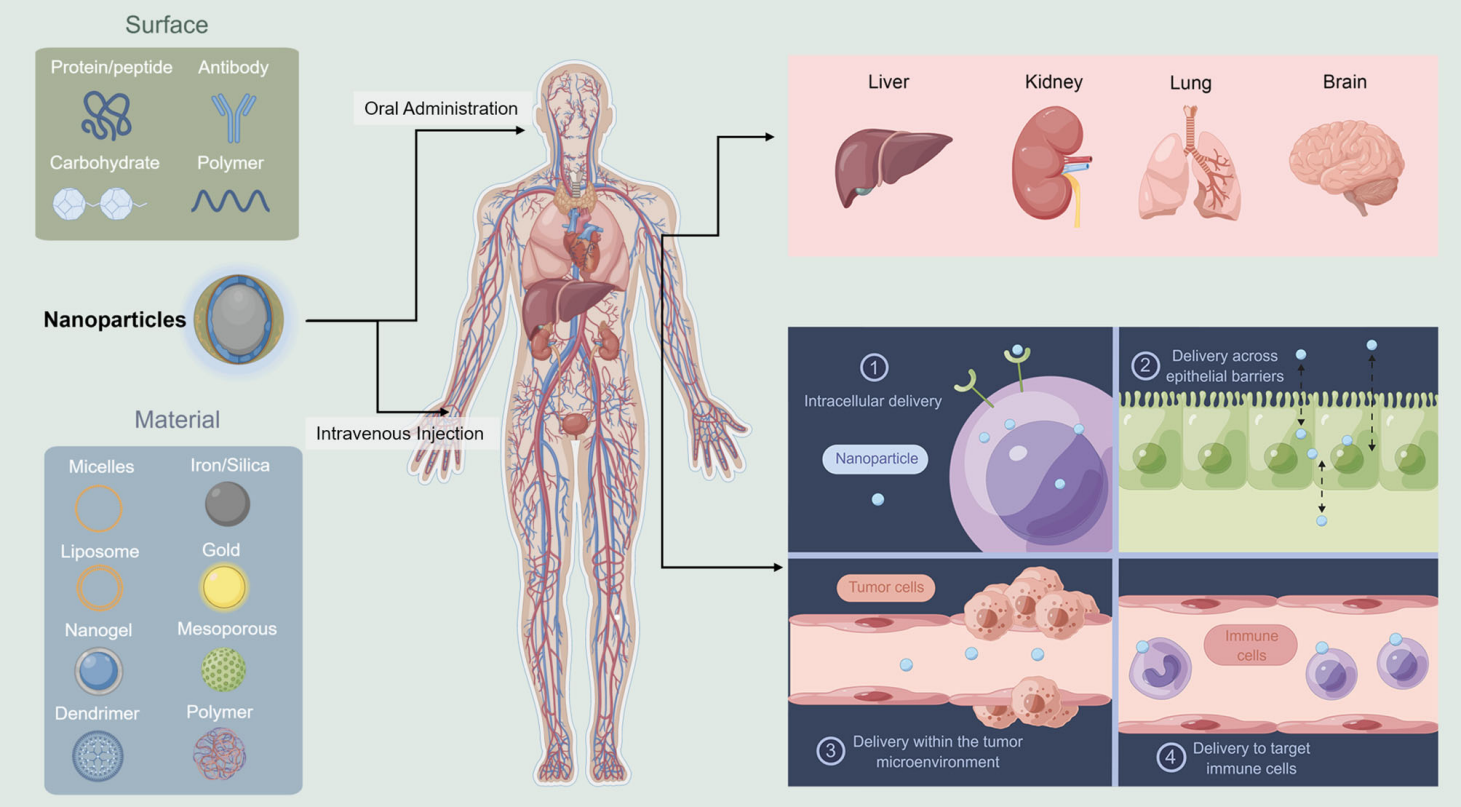
Schematic illustration of nanoparticles delivery system. Liver and kidney can filter most of the NPs. Different NPs are used in different administration ways (This figure was drawn by Figdraw).

Drug delivery is the process by which the medication from outside the body reaches the organs or cells (**Figure 1**). The reticuloendothelial system of the liver and kidney can filter most of the intravenously administered NPs (75). Most of the NPs are degraded, and few that are not degraded are retained. The Kupffer cells, which are phagocytic immune cells lining the liver sinusoids, are the key cells for the elimination of NPs (76). Actually, if the target system is reticuloendothelial system, the NPs can perfectly satisfy the demands since they can always reach the renal system effectively (77). While the NPs pass through the other organs, they are eliminated depending on their physical and biological features.

Once the NPs reach the target organs, they need to exit the vasculature and enter the targeted cells. They exit from the vasculature depends on the physiology of the vessel. In the liver, the NPs should be <100 nm (78), while in the kidney, it should be <6 nm (79). Transferring the NPs to the brain is even more difficult because of the blood–brain barrier (80). Extracellular matrix and connective tissue cells are tissue stroma, and these become barriers for NPs to enter the target cells. The cells absorb the particles in different ways, such as membrane fusion (81), caveolin-mediated endocytosis (82), clathrin-mediated endocytosis (83), micropinocytosis (84), or phagocytosis (85). The last step is when the NPs reach to the actual subcellular location for action. In this process, the challenge is how to escape the endosome.

There are two methods to increase the delivery efficiency: 1. Reduce the number of barriers by changing the route of administration. More barriers, more degradation. How to reach the destinated organs or cells with less elimination is of great concern. 2. Change the targeted position to make the delivery easier. For example, if the melanoma cells are hard to target, maybe we can aim to target the microenvironment easier. How to design the best NPs and the routes of administration are the two current areas of research advances in NPs and Melanoma.

**Figure 2:**
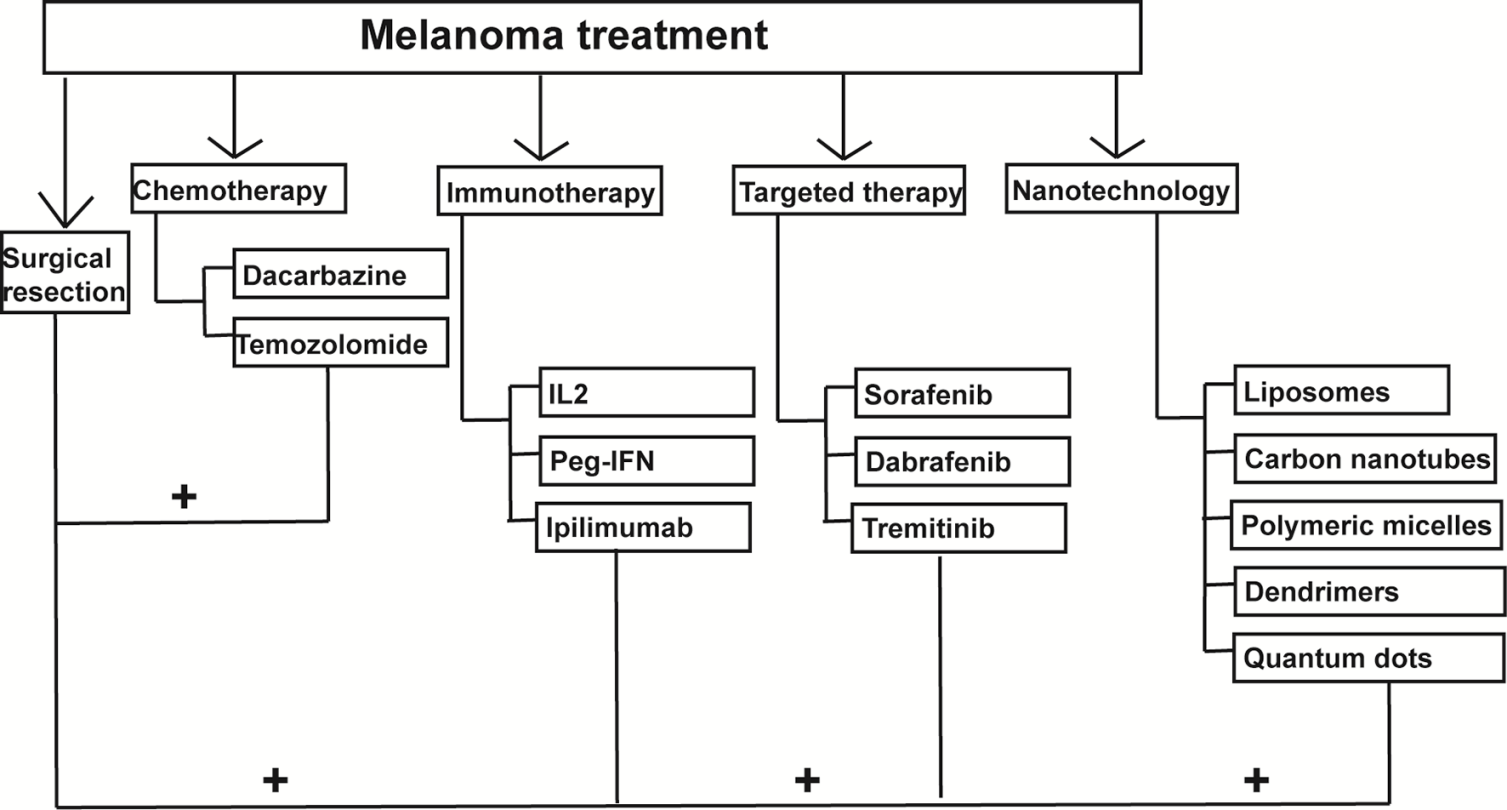
Nanoparticles-mediated combination therapies for melanoma treatment. Nanoparticles can be combined with chemotherapy, immunotherapy and photodynamic therapy to treat melanoma.

With conventional treatments, such as surgical resection, chemotherapy, radiotherapy, PDT, immunotherapy, or targeted therapy, chemotherapy drugs lack specificity for tumor sites, and melanoma cells often develop drug resistance to these drugs. Sometimes the drugs achieve minimal therapeutic effect but lots of side effects. Also, tumor-driven immunosuppression makes the immunotherapy hard to work and metastasis rate is still high (86). More and more researchers are trying to find new therapeutic approaches to treat melanoma. The aim is to target the melanoma cells more specifically with minimal adverse effects, using the small size and components of NPs (87). Liposomes and niosomes, polymeric NPs, inorganic particles, carbon nanotubes, and others are good vesicular carriers. The factors concerned in the melanoma NPs carriers included the core, targeting ligands, and the stimulation triggers of releasing the cargo (88). We discuss in the following sections, NPs in combination with chemotherapy, immunotherapy, or radiotherapy for the treatment of melanoma (**Figure 2**) (89).

### NPs and Chemotherapy Combined Treatment

Chemotherapy with DTIC is the standard treatment approved by the United States Food and Drug Administration. In a British study, the response rate to DTIC was 16%, with a 6-year survival rate of 31% (47). Monotherapy can achieve little in the treatment of melanoma, and even polytherapy cannot prolong the overall survival (89). Chemotherapy has a low response rate (12%) and combination with immune checkpoint inhibitor also cannot cure advanced melanoma (90). Adverse effects, less sensitivity, high toxicity, and drug resistance are the most common challenges associated with chemotherapy.

NPs are used as carriers to deliver drugs to the intended cells in the organs. NPs reduce systemic toxicity and degradation through the delivery process (87, 91). Meanwhile, NPs have the preferential accumulation in the melanoma, owing to the enhanced EPR (permeation and retention effect), which could promote their cellular uptake. They can regulate the pH, temperature, and responsiveness of enzymes due to their physical characteristics. They can pass easily through the vessels because of their small size and reach the targeted cells. NPs can also increase the concentration of the chemotherapy drugs in the intended organs. There are two mechanisms by which NPs deliver the drugs to the targeted cells: passive and active targeting. In passive targeting, NPs can increase the permeability and retention of drugs in the designated organs. In active targeting, NPs increase the accuracy of targeting and uptake of the drug by increasing the ability of ligands to recognise the tumour cell receptors.

There are many successful investigations with the combination of NPs and chemotherapy. The drug doxorubicin encoded by carbon nanotubes increased cell death (90%) by inducing a moderate G2-M phase arrest (17.7 ± 1.1%) and reduced tumour size in mice bearing B16–F10 melanoma (92). Another group of researchers tried to encode doxorubicin with chitosan/alginate NPs and showed a lower rate of release of doxorubicin, better transport, and higher intracellular concentration (93) due to the sustained release of the drug which provided better accumulation and longer cytotoxic effect of encapsulated doxorubicin in melanoma cell lines. More and more NPs were developed to encode chemotherapeutic agents. NPs/10-Hydroxycamptothecin encapsulated by NPs was designed to enhance the tumour penetration capability for chemotherapy. The proliferation of melanoma cell lines (B16F10 and B16F1) decreased, and apoptosis increased with this treatment (94). The tumour growth was also inhibited compared to that seen in PBS (Phosphate-buffered saline) injected tumour-bearing mice. This effect was considers derived from the positive surface of NPs/HCPT enhanced cells internalization through endocytosis. A new nanoformulation of paclitaxel-loaded cholic acid-functionalised star-shaped poly(lactide-co-glycolide) (PLGA)-d-α-tocopheryl polyethylene glycol 1000 succinate (TPGS) NPs (PTX-loaded CAPLGA-TPGS NPs) was produced by Yongsheng et al. to treat malignant melanoma, and it showed a higher rate of release rate (78.69%), higher cellular uptake efficiency, and reduced growth of tumours in mice compared to those treated with saline (95). This year, DTIC (Dacarbazine)-NPs(Cholic acid -poly (lactide-co-glycolide)-b-polyethylene glycol)-Apt(nucleic acid aptamer AS1411) was developed to treat melanoma cell lines and showed good toxicity to MM but few side effects (96). The combination of NPs and chemotherapy have increased the EPR of the chemotherapy drug although different mechanisms.

### Combined Treatment Using NPs and Immunotherapy

Immunotherapy aims to enhance or inhibit the immune system and can be classified as active and passive immunotherapy (97), depending on the method of boosting the immune response i.e., using an antigen or the intrinsic immune response itself. However, immunotherapy can cause many adverse effects, like attacking healthy organs, changing the immune system, or resistance (98). The rate of primary and secondary resistance to immunotherapy drugs like checkpoint inhibitors is increasing every year.

NPs help in enhancing the effect of immunotherapy in melanoma through different mechanisms (86, 99): 1. As the tumour cells inhibit the immune response, the accumulation of NPs at the site of the primary tumour can relieve the immunosuppressive effect and help the immune system recognise the tumour cells and kill them. Cancer immunoediting is a process that targets the microenvironment and enables the melanoma cells to escape the killing effect of the immune system. NPs try to target the macrophages and NK cells to boost the immune system, inhibit tumour growth, and prevent recurrence. NPs can also induce tumour immunogenic cell death or generate more tumour-associated antigens. 2. NPs can target the peripheral immune cells to inhibit tumour growth or mimic the artificial immune cells or improve the efficacy of adoptive T cells. 3. NPs prevent the formation of the pre-metastatic niche by reducing inflammation and myeloid-derived suppressor cells. 4. Prevent recurrence by directly applying the NPs on the surgical wounds.

Many NPs are under investigation for clinical use. Nanovaccine is one option. DGBA-OVA-CpG (Guanidinobenzoic acid-ovalbumin-cytosine-guanine dinucleotides) nanovaccine increased the antigen-presenting cells such as dendritic cells *in vitro* and *in vivo.* It also increased the generation of circulating interferon-γ+ CTLs (Cytotoxic T-lymphocytes) 4 times compared to that in the control group. The combination of DGBA-OVA-CpG nanovaccine with anti-PD-1 blockade significantly reduced the growth of melanoma in mice compared to that in the control group (by more than 50%), and the survival rate increased by nearly 37.5% (100). It raised great interest for researchers to develop vaccines constructed by dendrimers capable of cytosol delivery of proteins to treat melanoma. A hydrogel system was used to deliver celecoxib and PD-1 blockade to treat B16-F10 melanoma in mice to increase the antitumour effect of PD-1 blockade. This method decreased the tumour size to nearly 10% compared to that in the control group and increased the survival in mice (101). Synergistically delivery both celecoxib and PD-1 from this hydrogel system enhanced the presence of CD4^+^ T cells and CD8^+^ T cells in the immune system and finally formed an impaired pro-tumor angiogenic and inflammatory microenvironment.

### NPs and Photodynamic Therapy (PDT) Combined Treatment

PDT promotes the accumulation of photosensitisers (PSs) activated by irradiation in the targeted organs or cells and kills the disease cells through ROS generated by photosensitizers. Permeability and retention are the most important challenges in conventional photodynamic therapy. Although PS is not recommended by the guidelines, it is still a useful adjuvant therapy, and many researchers have attempted to increase the efficacy of PS. NPs, which have a smaller size, can increase the permeability, target the cells more precisely and reduce the degradation of PDT. NPs can increase the solubility of PSs in water and thus increase their absorption rate. Furthermore, NPs reduce the elimination of PSs and increase the retention time. NPs, due to their small size and physical characteristics, make the penetration of PSs to the targeted cells easier. As diseased cells can adapt their own oxidative system and develop resistance to the treatment, NPs help overcome this challenge through high ROS (Reactive oxygen species) production.

5-aminolaevulinic acid-mediated photodynamic therapy was used to treat the A375 melanoma cell lines and showed decreased survival and increased apoptosis (102). 5-ALA caused the accumulation of endogenous photosensitizer protoporphyrin IX (PpIX) in the mitochondria of cells, thus increasing the apoptosis of melanoma cells and the mechanism of this can contribute to improve the therapeutic efficacy. Zhao et al. tried to encapsulate phthalocyanine 4 using silica NPs to treat different melanoma cell lines and showed higher permeability and lower cell survival than controls (103). Modifying the surface of silicon nanoparticles to encapsulate the photosensitizers and the antibodies specific to melanoma cells can be a new method in melanoma treatment. Yttrium oxide NPs were also used in combination with X-rays to treat melanoma cells. Porosnicu found that it increased the production of ROS,thus influenced the integrity of the mitochondria and increased the DNA damage (104). Magnetic NPs and albumin-stabilised paclitaxel NPs are also under investigation for combination therapy.

There are some clinical trials about the combination of NPs and PDT. Verteporfin with laser irradiation was the most used combination, and it reduced tumour growth with less regression; however, more data of the clinical trials is necessary.

## Conclusions and Perspectives

The incidence of melanoma is increasing rapidly, and several patients develop distant metastasis. The main cause of melanoma is excess UV light exposure. Although surgical removal is the core strategy to treat melanoma, adjuvant therapies, including chemotherapy, immunotherapy, PDT therapy are also combined with surgery. Due to the low permeability, short retention, and high resistance to the existing therapeutic agents, new methods to treat melanoma are urgently needed.

NPs due to their unique physical features, are under investigation. Combinations of liposomes, carbon nanotubes, polymeric micelles, dendrimers and quantum dots, and other technologies with conventional adjuvant therapies are being attempted to deliver the drug more efficiently, reduce the degradation and toxicity, and target the intended cells more accurately. Although considerable progress has been achieved in the previous years, agents with further increased specificity, smaller size, and lower toxicity are needed and the technologies to combine them with conventional medications are still necessary. Moreover, clinical trials should be initiated to test the NPs to increase their use in clinics.

## Author Contributions

All authors contributed to the design of the study and writing of the manuscript. HC, KH, and JY undertook the research. KH, HC, and XC wrote the main manuscript text and prepared figures. LW and XC revised the article critically for important intellectual content and final approval of the version to be submitted. JY responded to the reviewers’ questions and revision manuscript preparation. All authors reviewed the manuscript.

## Funding

This study was financially support by the foundation from the National Natural Science Foundation of China (No.81873630).

## Conflict of Interest

The authors declare that the research was conducted in the absence of any commercial or financial relationships that could be construed as a potential conflict of interest.

## Publisher’s Note

All claims expressed in this article are solely those of the authors and do not necessarily represent those of their affiliated organizations, or those of the publisher, the editors and the reviewers. Any product that may be evaluated in this article, or claim that may be made by its manufacturer, is not guaranteed or endorsed by the publisher.

## Abbreviations

AM, Acral melanoma; BRAF, v-raf murine sarcoma viral oncogene homolog B1; CTLs, Cytotoxic T-lymphocytes; CM, Cutaneous melanoma; DGBA-OVA-CpG, Guanidinobenzoic acid-ovalbumin-cytosine-guanine dinucleotides; EPR, Permeation and retention effect; UV, Ultraviolet; PDT, Photodynamic therapy; NPs, Nanoparticles; PSs, Photosensitisers; TWT, Triple wild type; MM, Mucosal melanoma; MAPK, The mitogen-activated protein kinase; MEK, Mitogen-activated protein kinase kinase; VEGF, Vascular endothelial growth factor; PBS, Phosphate-buffered saline; ROS, Reactive oxygen species; UVB, Ultraviolet B-rays; RAS, Rat sarcoma virus; NF1, Neurofibromatosis 1; UM, Uveal melanoma; DTIC, Dacarbazine.
